# Frontal Fibrosing Alopecia Quality of Life Index: A Validated Disease-Specific Questionnaire Involving Women

**DOI:** 10.3390/jcm12030824

**Published:** 2023-01-20

**Authors:** María Librada Porriño-Bustamante, Trinidad Montero-Vílchez, María Antonia Fernández-Pugnaire, Salvador Arias-Santiago

**Affiliations:** 1 Dermatology Department, University Hospital La Zarzuela, 28023 Madrid, Spain; 2 Dermatology Department, University of Granada, 18016 Granada, Spain; 3 Dermatology Department, University Hospital Virgen de las Nieves, 18014 Granada, Spain; 4 Dermatology Department, University Hospital San Cecilio, 18016 Granada, Spain; 5 School of Medicine, Institute of Biosanitary Investigation ibs, Granada University, 18016 Granada, Spain

**Keywords:** frontal fibrosing alopecia, scarring alopecia, quality of life

## Abstract

Quality of life (QoL) can be affected in patients with alopecia. The few studies that evaluate QoL in FFA use unspecific questionnaires. The aim of this report was to design and validate a specific questionnaire to assess the impairment of QoL in FFA patients. A specific questionnaire, called the Frontal Fibrosing Alopecia Quality of Life Index (FFA-QLI), was designed and validated using the Dermatology Life Quality Index (DLQI). One-hundred and one women with FFA were included. Cronbach’s alpha value was 0.865, and the intraclass correlation coefficient between all the items in the questionnaire was 0.870. The FFA-QLI correlated positively with the DLQI (r = 0.729, *p* < 0.001). Patients with severe FFA showed a higher FFA-QLI (19.72) score compared to those with a mild disease (14.11) (*p* = 0.002), and the area under the curve for identifying severe disease was greater in the FFA-QLI than in the DLQI. The cut-off points were used to select patients with mild, moderate, and severe impairment in QoL. A score < 21 in the FFA-QLI corresponded to a low impact on QoL; values > 35 matched with greater QoL impairment; and values ranging from 21 to 35 corresponded to moderate QoL alteration. To conclude, a validated disease-specific questionnaire to assess QoL in FFA patients is here presented, with a greater power to discriminate severe cases of FFA than the DLQI.

## 1. Introduction

The prevalence of frontal fibrosing alopecia (FFA) has been increasing progressively since its first description [1], becoming the most common type of scarring alopecia [2].

As hair is an important element of identity and self-image, alopecia can impact negatively on quality of life (QoL). QoL is generally assessed by unspecific self-reporting questionnaires. It has been demonstrated that alopecia may have substantial psychological consequences, especially in women [3]. Feelings of loss of self-confidence, low self-esteem, and heightened self-consciousness are common feelings [4]. Furthermore, people with alopecia are more likely to have depression and anxiety [4,5]. Generic questionnaires may not detect all patients with QoL disorders, so for some specific diseases, a specific questionnaire may provide a more appropriate assessment, as happens in hidradenitis suppurativa, alopecia areata, or androgenetic alopecia (AGA) [6,7,8].

Most studies regarding QoL and alopecia are about alopecia areata and AGA [7,9]. However, there are few reports regarding scarring alopecia and QoL, and even fewer about FFA. All of them used pre-existent general scales, such as the Illness Perception Questionnaire, the Hospital Anxiety and Depression Scale (HADS), and the Dermatology Life Quality Index (DLQI) [10,11,12,13,14,15]. The DLQI is a 10-item self-reported measure of QoL, (scored from 0 to 30), in patients with dermatological conditions, in which scores 0–1 indicate that QoL is not affected, 2–5 identify mild QoL impairment, 6–10 reflect a moderate impact, 11–20 a severe impact, and > 21 a very severe QoL impairment [13]. HADS is a 14-item self-reported measure of anxiety (HADS-A, 7 items) and depression (HADS-D, 7 items); the sub-scales range from 0 to 21, and scores < 7 suggest a non-case, 8–10 a probable case, and > 11 a definite case [14].

The main objective of this study was to design and validate a new disease-specific questionnaire for FFA, the Frontal Fibrosing Alopecia Quality of Life Index (FFA-QLI), for QoL measurement in patients with FFA.

## 2. Materials and Methods

A cross-sectional study, including women with FFA and a control group, was performed at the Dermatology Departments of the University Hospital San Cecilio and Virgen de las Nieves, both situated in Granada, Spain. Inclusion criteria for FFA patients were the presence of frontal and/or temporoparietal hairline recession, leading to a scarring band without follicular openings, with or without perifollicular erythema and follicular hyperkeratosis. Inclusion criteria for the control group were as follows: women aged between 45 and 95 years, without any hair disease. Control individuals were people who had consulted the Dermatology Department for other reasons (naevi, seborrheic keratosis, etc.). The exclusion criterion for both groups was the male sex. All patients and control subjects signed an informed consent, and the project was approved by the local ethics committee.

Demographic and general information was recorded for both patients and control subjects. In patients, data regarding the alopecia were obtained, such as age of onset, presence of perifollicular erythema and follicular hyperkeratosis, presence of itchiness or trichodynia, eyebrow and eyelash alopecia, and the existence of facial papules and other types of alopecia. The severity grade was assessed following the V-grade classification described by Vañó et al. and grouped into mild (I-II) or severe (III-V) FFA [16].

Three types of questionnaires were administered to both patients and control subjects: the DLQI, the HADS, and the newly designed FFA-QLI. A translated English version of the questionnaire is provided (Supplementary Table S1), although the validated version is the Spanish one (Supplementary Table S2).

The FFA-QLI consisted of 20 questions divided into three parts: Items 1 to 7 are questions related to emotions; Items 8 to 14 are questions about their social life; and Items 15 to 20 are questions about functional changes. The responses were scored from 0 (not affected at all) to 3 (highly affected). The results of the FFA-QLI are presented on a scale varying between 0 (best QoL) and 60 (worst QoL). The questions were developed by dermatologists working in the trichology area. They were selected on the basis of the main clinical signs and symptoms of FFA, focusing on different areas of life in which patients can feel ashamed or worried and also on behaviors that could be modified because of the alopecia. The questionnaire was refined after interviewing 25 patients, and the questions were modified to improve the patients’ understanding of them, as a result of the feedback from the first impressions of the interviewed patients; after that, the final questionnaire was used for all the participants.

The psychometric validation of the FFA-QLI was based on the DLQI and the HADS. Reliability was evaluated using internal consistency analysis with the Cronbach α (acceptable if > 0.7) and reproducibility analysis with the intraclass correlation coefficient (ICC) (adequate if > 0.7). To determine the test–retest reliability, the ICC for the global value of the questionnaire and Cohen’s kappa (acceptable strength of agreement if kappa > 0.5) of the items were calculated from the original FFA-QLI.

Convergent validity, examining the degree to which two measures of the constructs are related, was assessed by calculating the extent of correlation between raw scores from the FFA-QLI and the DLQI using the Pearson correlation coefficient. The cut-off points to select patients with mild, moderate, and severe QoL impairment were calculated using receiver operating characteristic (ROC) curve analysis and comparison with the DLQI categories.

Continuous data are presented as the mean (standard deviation) and categorical data as the relative (absolute) frequency. Continuous data were tested for normality using the Kolmogorov–Smirnov test. Student’s *t*-test was applied to compare the mean values of the quantitative variables. Qualitative variables were analyzed with the χ^2^ test. Statistical significance was defined by a two-tailed *p* < 0.05. SPSS Version 24.0 (SPSS Inc, Chicago, IL, USA) was used for the statistical analyses.

Regarding the sample size, at least four participants per variable would be necessary to validate a questionnaire [17]. As the FFA-QLI consists of 20 questions, a total of 20 variables were evaluated, requiring at least 80 participants.

## 3. Results

A total of 101 women with FFA and 40 healthy women were included. Case and control groups were comparable regarding age (63.45 vs. 63.05 years, respectively; *p* = 0.824) and sex. Additional data about patients are listed in Table 1.

The mean FFA-QLI score in patients was 17.11 (SD 9.37) (ranging from 3 to 44), whereas in control subjects, it was 0.98 (SD 1.31) (ranging from 0 to 5) (*p* < 0.001). The mean DLQI score in patients was 3.42 (SD 3.65), compared to 0.50 (SD 0.99) in control individuals (*p* < 0.001). The mean HADS-A score in patients was 7.91 (SD 4.02), while in control participants, it was 5.65 (SD 3.37) (*p* = 0.002). Finally, the mean HADS-D score in patients was 4.6 (SD 3.39), compared to 4.25 (SD 3.24) in control individuals (*p* = 0.57).

Cronbach’s α value was 0.911 in the overall cohort, 0.865 in patients with FFA, and 0.324 in healthy individuals. The ICC between all the items in the questionnaire was 0.912 in the overall cohort, 0.870 in patients with FFA, and 0.324 in healthy individuals. Regarding convergent validity, the FFA-QLI was positively correlated with the DLQI (r = 0.729, *p* < 0.001) and HADS (r = 0.361, *p* < 0.001) in patients with FFA.

**Figure 1:**
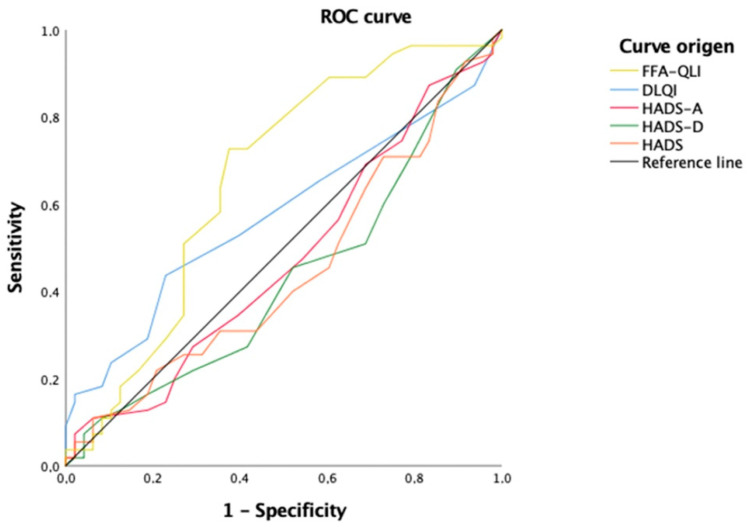
Receiver operating characteristic (ROC) curve to assess discriminative power differences between scales to select severe FFA. The FFA-QLI showed higher discriminative power to discriminate patients with severe FFA, compared to all the other questionnaires, the DLQI, HADS, HADS-A, and HADS-D.

According to Vañó et al.’s classification, 46.5% (47/101) had a mild disease and 53.5% (54/101) had a severe disease. Patients with a severe disease showed higher values in the FFA-QLI (19.72 vs. 14.11, *p* = 0.002) and DLQI (4.24 vs. 2.47, *p* = 0.011), without differences in the HADS scores. Moreover, it was observed that the FFA-QLI had a higher discriminative power to select severe FFA patients (area under the curve (AUC) = 0.704, *p* < 0.001) than the DLQI (AUC = 0.603, *p* = 0.076) (Figure 1).

**Figure 2:**
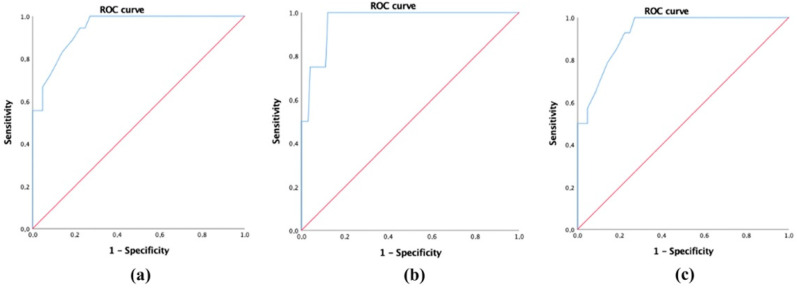
Receiver operating characteristic (ROC) curve to select cut-off points in the Frontal Fibrosing Alopecia Quality of Life Index (FFA-QLI) to split patients into (**a**) mild (< 21), (**b**) severe (> 35), or (**c**) moderately (21–35) impaired quality of life.

To select patients with mild, moderate, and severe QoL impairment, cut-off points were delimited by comparing the values for the FFA-QLI and DLQI. The DLQI was used because it yielded the best correlation values. The ROC curve analysis showed that values lower than 21 in the FFA-QLI corresponded to patients with low QoL impairment, with a sensitivity of 88.9% and a specificity of 80.7% (AUC = 0.942, *p* < 0.001) (Figure 2a). To select patients with greater QoL disorder, it was proposed that the cut-off point be 35, with a sensitivity of 75% and a specificity of 95.9% (AUC = 0.961, *p* = 0.002) (Figure 2b). The separation between the categories of mild and moderate was proposed at a cut-off point of 21, with a sensitivity of 85.7% and specificity of 80.7% (AUC = 0.929, *p* < 0.001) (Figure 2c).

The distribution of the scores of all the questionnaires in FFA patients is presented in Table 2. Patients with a more severe disease had significantly higher mean FFA-QLI scores in the following items: worrying about eyebrow (Item 5, *p* = 0.022) and hair loss (Item 6, *p* = 0.05), using a different hairstyle to cover the alopecia (Item 16, *p* = 0.001), capacity to forget the alopecia (Item 18, *p* = 0.005), use of eyebrow make-up or having the eyebrows micropigmented (Item 19, *p* = 0.003), and use of wigs or any type of hair system (Item 20, *p* = 0.032).

The test–retest reliability was tested in 30 patients with FFA. The mean FFA-QLI was similar in the first and the second test (17.48 vs. 16.86). The time between the test and retest was 30 days. The test–retest reliability was high (ICC = 0.989). The correlation between items in the two tests was also high. Moreover, Cohen’s kappa was > 0.5 (*p* < 0.001) between each item in the first and second questionnaire, showing at least an acceptable strength of agreement.

**Figure 3:**
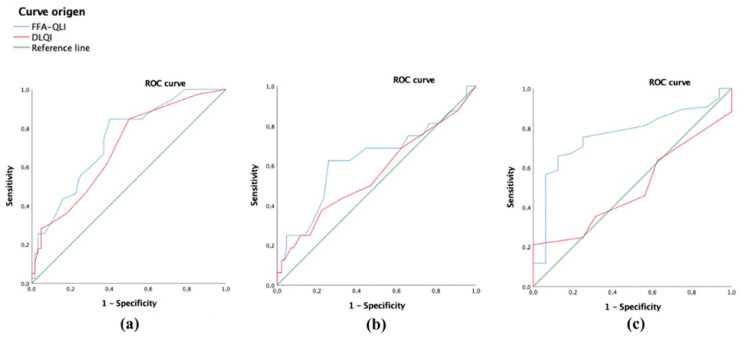
Receiver operating characteristic (ROC) curve to assess discriminative power differences between scales to select (**a**) long-lasting cases of FFA, (**b**) patients with facial papules, and (**c**) patients with eyebrow alopecia. The FFA-QLI showed higher discriminative power than the DLQI to discriminate patients with long-lasting FFA (**a**) and eyebrow alopecia (**c**). However, although the FFA-QLI had more power to select patients with facial papules than the DLQI (**b**), it did not have enough power to do so.

No differences in the FFA-QLI scores were noted between patients who had pruritus or trichodynia and those who did not have them nor regarding the age of the patients. The mean score of the FFA-QLI was higher in those with an earlier debut of the alopecia (< 58 years) compared to those with a later age of onset (> 58 years) (mean scores 18.9 vs. 15.28, respectively, *p* = 0.052). Patients with a long-lasting alopecia (> 36 months) had higher FFA-QLI scores (mean scores 21.12 vs. 13.33, *p* = 0.001); the FFA-QLI had a higher discriminative power to select patients with long-lasting alopecia (AUC = 0.739, *p* < 0.001) compared to the DLQI (AUC = 0.696, *p* = 0.001) (Figure 3a). With regard to extrascalp involvement, patients with facial papules had higher FFA-QLI mean scores (21.5 vs. 16.28, *p* = 0.04), although the FFA-QLI did not have enough power to select patients with facial papules (AUC = 0.629, *p* = 0.104), nor did the DLQI (AUC = 0.557, *p* = 0.468) (Figure 3b).

Patients with eyebrow alopecia had higher FFA-QLI scores (mean value 18.29 vs. 10.81, *p* < 0.001); the FFA-QLI had a higher discriminative power to select patients with eyebrow alopecia (AUC = 0.766, *p* = 0.001) compared to DLQI (AUC = 0.496, *p* = 0.963) (Figure 3c). No significant differences were found in the FFA-QLI scores in patients regarding eyelash alopecia (19.26 with vs. 16.32 without it, *p* = 0.165) or in relation to the presence of other types of alopecia (18.57 with vs. 15.98 without them, *p* = 0.170). No differences were noted in the FFA-QLI scores regarding the occupational status (active vs. inactive), civil status (living alone vs. married), and having children (yes vs. no).

## 4. Discussion

In this study, a specific and validated questionnaire to assess QoL in patients with FFA is proposed, called the FFA-QLI. It has demonstrated a higher capacity than the DLQI to select patients with severe FFA and also with long-lasting FFA and eyebrow alopecia, features that were related to a worse FFA-QLI score in FFA patients.

Around a third of patients with FFA showed at least a moderate impairment of their QoL after evaluation with the FFA-QLI. Women’s QoL is usually more affected than men’s [3], and this may be related to the fact that male alopecia is socially more acceptable, and even considered normal, compared to alopecia in women. One study found that patients with primary cicatricial alopecia experienced significant psychological distress and impaired QoL, both of which were associated with key beliefs about illness [10]. In addition, in lichen planopilaris (LPP), higher disease activity was correlated with depression.

Only three reports regarding the assessment of QoL in specifically FFA patients have been published. Saceda-Corralo et al.’s study included 82 FFA female patients and used the DLQI, the HADS, and the Revised Illness Perception Questionnaire [11]. They found a negative association between QoL and FFA, without association between QoL and the alopecia severity. Other findings were that older patients had worse scores in the HADS (this means being more likely to have anxiety or depression), that patients with severe alopecia appeared to feel powerless to control their illness, and that trichodynia was related to impaired QoL. Valesky et al.’s study included 12 female patients with FFA and found that the QoL of their patients was good (but not excellent) and no significant correlation between QoL and the duration of the disease or maximal hairline regression [12]. They concluded that there might be a slightly negative correlation between FFA and QoL. On the other hand, during the validation of the FFA severity score, Saceda-Corralo et al. found no correlation between the severity index and QoL [18]. In the third article, Doche et al. compared the disease activity in LPP (n = 10) and FFA (n = 27) using the LPP Activity Index (LPPAI) with the score in the DLQI, and found no significant association between them in both groups [15]. However, they noted that patients with LPP and FFA with at least one associated non-scalp lesion, tended to have a higher DLQI score and, consequently, a poor QoL.

All the questionnaires used in these studies were unspecific, as the DLQI is used for different types of skin conditions and the HADS is even more general. FFA has very characteristic features, such as eyebrow alopecia, facial papules, and the typical scarring alopecic band, which may specifically affect the QoL of patients. Therefore, to obtain an accurate assessment of QoL impairment in patients with FFA, a specific questionnaire is necessary. The proposed FFA-QLI showed a close correlation with the DLQI and a poor correlation with the HADS, meaning that the FFA-QLI is more valuable in assessing the global impairment in QoL than evaluating anxiety or depression.

Most of our patients had mild alteration in their QoL, similar to previous reports [12]. However, more than a third of patients showed moderate or severe detriment in their QoL; the higher frequency compared to previous reports may be due to the use of a more specific questionnaire. Furthermore, FFA-QLI scores were higher in patients with severe disease, and FFA-QLI demonstrated a high power to select FFA patients with severe disease, something that the DLQI did not show according to previous studies [11,12]. The presence of symptoms was not associated with higher total scores in FFA-QLI, neither was the age of patients. Nevertheless, patients with a longer duration of their alopecia showed higher scores in the FFA-QLI, which may be due to a greater accumulation of psychological tiredness. Patients with facial papules and with eyebrow alopecia had a greater FFA-QLI score, in accordance with previous reports, which found a poor QoL in patients with at least one associated non-scalp lesion [15]. Interestingly, the social environment of patients, regarding marital status, motherhood, or occupational status, was not related to any differences in the FFA-QLI.

Limitations to the present study include possible cultural bias related to the monocultural development of the questionnaire. Nevertheless, this is a potential limitation inherent in most questionnaire development processes. FFA is, by far, more common in women, whose QoL is usually more impaired than the QoL in male patients with alopecia. In this article, the FFA-QLI has been validated only in women with FFA.

## 5. Conclusions

In conclusion, a specific validated questionnaire for FFA is proposed, called the FFA-QLI. It also shows a higher power to discriminate patients with a more severe disease than the DLQI. The impact of FFA on QoL could be higher than that which was previously reported using only unspecific questionnaires. This questionnaire may help dermatologists identify patients with a greater impairment in their QoL and seek help for the patients who need it.

## 6. Patents

Validated questionnaire registered in safeCreative, with the number 2205301246067.

## Figures and Tables

**Table 1:** Additional data about FFA patients.

N = 101	Mean Values (SD) and Frequencies
Female sex	100%
Age (years)	63.45 (SD 9.32)
Marital status - Single, divorced, widowed - Married	31.7% (32/101) 68.3% (69/101)
Occupational status - Active - Inactive (retired, unemployed)	32.7% (33/101) 67.3% (68/101)
Children (yes)	90.1% (91/101)
Menopause (yes)	89.1% (90/101)
Age of menopause (years)	50.38 (SD 3.87)
Age of onset of FFA (years)	58.53 (SD 9.66)
Pruritus (yes)	75.2% (76/101)
Trichodynia (yes)	18.8% (19/101)
Grade of FFA - Grade I - Grade II - Grade III - Grade IV - Grade V	4% (4/101) 42.6% (43/101) 33.7% (34/101) 10.9% (11/101) 8.9% (9/101)
Long-lasting alopecia (>36 months) (yes)	48.5% (49/101)
Eyebrow alopecia (yes) - Partial - Total	84.2% (85/101) 49.5% (50/101) 34.7% (35/101)
Eyelash alopecia (yes)	26.2% (27/101) (all partial)
Facial papules (yes)	15.8% (16/101)
Perifollicular erythema (yes)	85.1% (86/101)
Follicular hyperkeratosis (yes)	93.1% (94/101)
Other alopecias (yes) - AGA - LPP - AA - TA	43.6% (44/101) 33.7% (34/101) 7.9% (8/101) 1% (1/101) 1% (1/101)

AGA: androgenetic alopecia; LPP: lichen planopilaris; AA: alopecia areata; TA: tractional alopecia; SD: standard deviation.

**Table 2:** Distribution of the scores of the questionnaires in FFA patients.

QoL Impairment	FFA-QLI	DLQI
No impairment	0	36.6% (37/101)
Mild impairment	68.3% (69/101)	45.5% (46/101)
Moderate impairment	24.8% (25/101)	13.9% (14/101)
Severe impairment	6.9% (7/101)	3% (3/101)
Very severe impairment	-	1% (1/101)
	**HADS-A**	**HADS-D**
Non-case	48.5% (49/101)	82.2% (83/101)
Probable case	28.7% (29/101)	11.9% (12/101)
Definite case	22.8% (23/101)	5.9% (6/101)

## Data Availability

Not applicable.

## Supplementary Materials

The following Supporting Information can be downloaded at: https://www.mdpi.com/article/10.3390/jcm12030824/s1, Table S1: Frontal Fibrosing Alopecia Quality of Life Index (FFA-QLI). English version (non-validated); Table S2: Alopecia Frontal Fibrosante Índice de Calidad de Vida (AFF-ICV) (original version of the validated questionnaire).
