# Supplementary material for: Frontal Fibrosing Alopecia Quality of Life Index: A Validated Disease-Specific Questionnaire Involving Women
Source: J Clin Med. 2023 Jan 20;12(3):824. doi: 10.3390/jcm12030824 (PMC9917648; doi:10.3390/jcm12030824)
Supplement: Supplementary file 1 [file jcm-12-00824-s001.zip › jcm-2128364-supplementary.pdf]

Supplementary Materials:

Table S1. Frontal Fibrosing Alopecia Quality of Life Index (FFA-QLI). English version (non-validated);

**Emotional**
1. Do you feel like you are not the same person because of having lost your eyebrows?<br>0. No. / I have not lost my eyebrows.<br>1. Yes, a little.<br>2. Yes, a lot.<br>3. Yes, very much.
2. Have you lost your self-confidence?<br>0. Not at all.<br>1. Yes, a little.<br>2. Yes, a lot.<br>3. Yes, very much.
3. Would you prefer to suffer from an internal disease, more serious but not visible, instead of alopecia?<br>0. Not at all.<br>1. Yes, a little.<br>2. Yes, a lot.<br>3. Yes, very much.
4. Do you feel itchy or pain in your scalp?<br>0. Not at all.<br>1. Yes, a little.<br>2. Yes, a lot.<br>3. Yes, very much.
5. Are you worried about eyebrow loss?<br>0. Not at all.<br>1. Yes, a little.<br>2. Yes, a lot.<br>3. Yes, very much.
6. Are you worried about scalp hair loss?<br>0. Not at all.<br>1. Yes, a little.<br>2. Yes, a lot.<br>3. Yes, very much.
7. Do you think that your alopecia alters your body image?<br>0. Not at all.<br>1. Yes, a little.<br>2. Yes, a lot.<br>3. Yes, very much.
**Social**
8. Do you feel that your look interferes with your work?<br>0. Not at all. / No, I am already retired.<br>1. Yes, a little.<br>2. Yes, a lot.<br>3. Yes, very much.
9. Have you stopped going to the hairdresser's or reduced the frequency you go there because of the alopecia?<br>0. Not at all.<br>1. Yes, a little.<br>2. Yes, a lot.<br>3. Yes, very much.
10. Do you feel that people look at you (stare at you) because of your alopecia?<br>0. Not at all.<br>1. Yes, a little.

2. Yes, a lot.
3. Yes, very much.

11. Have you given up activities that you used to do because of your alopecia (leisure, sport,…)?
    0. Not at all.
    1. Yes, a little.
    2. Yes, a lot.
    3. Yes, very much.

12. Do you feel that your alopecia interferes with your sex life?
    0. Not at all.
    1. Yes, a little.
    2. Yes, a lot.
    3. Yes, very much.

13. Do you feel that your alopecia interferes with your relationship with your partner?
    0. Not at all.
    1. Yes, a little.
    2. Yes, a lot.
    3. Yes, very much.

14. Do you feel that your alopecia interferes with your relationship with your friends?
    0. Not at all.
    1. Yes, a little.
    2. Yes, a lot.
    3. Yes, very much.

Functional

15. Do you waste time trying to hide your alopecia (eyebrows or scalp)?
    0. Not at all.
    1. Yes, a little.
    2. Yes, a lot.
    3. Yes, very much.

16. Do you comb your hair differently than usual to hide your alopecia?
    0. Not at all.
    1. Yes, a little.
    2. Yes, a lot.
    3. Yes, very much.

17. Have you changed the frequency of combing or washing your hair because of fear of increasing your hair loss?
    0. Not at all.
    1. Yes, a little.
    2. Yes, a lot.
    3. Yes, very much.

18. Do you forget about your alopecia at any moment?
    0. Yes, a lot of the time.
    1. Yes, quite often.
    2. Yes, but rarely.
    3. No, never.

19. Do you make-up your eyebrows to hide the alopecia or have them tattooed?
    0. No. / No, I have not lost my eyebrows.
    1. Yes, I use make-up on them occasionally.
    2. Yes, I use make-up on them quite often.
    3. Yes, I use make-up on them everyday. / Yes, I have eyebrow tattoos to cover the eyebrow alopecia.

20. Do you use any type of wig or capilar prothesis to hide your alopecia?
    0. No, never.
    1. Yes, ocassionaly.
    2. Yes, quite often.
    3. Yes, always.

Score: mild impairment (< 21), moderate impairment (21-35), severe impairment (> 35)

Table S2. Alopecia Frontal Fibrosante Índice de Calidad de Vida (AFF-ICV) (Original version of the validated questionnaire)

**Emocional**
1. ¿Siente que no es usted mismo/a al haber perdido las cejas?<br>0. No. / No las he perdido.<br>1. Sí, un poco.<br>2. Sí, bastante.<br>3. Sí. Mucho.
2. ¿Ha perdido la confianza en usted mismo/a?<br>0. No.<br>1. Sí, un poco.<br>2. Sí, bastante.<br>3. Sí, mucho.
3. ¿Preferiría padecer un problema de salud interno (más importante, pero no visible, como diabetes, hipertensión,…) en lugar de la alopecia?<br>0. No.<br>1. Sí, un poco.<br>2. Sí, bastante.<br>3. Sí, mucho.
4. ¿Tiene picor o dolor en el cuero cabelludo?<br>0. No, nada.<br>1. Sí, un poco.<br>2. Sí, bastante.<br>3. Sí, mucho.
5. ¿Le preocupa la pérdida de las cejas?<br>0. No, nada.<br>1. Sí, un poco.<br>2. Sí, bastante.<br>3. Sí, mucho.
6. ¿Le preocupa la pérdida de cabello?<br>0. No, nada.<br>1. Sí, un poco.<br>2. Sí, bastante.<br>3. Sí, mucho.
7. ¿Piensa que la alopecia altera su imagen corporal?<br>0. No, nada.<br>1. Sí, un poco.<br>2. Sí, bastante.<br>3. Sí, mucho.
**Social**
8. ¿Piensa que su alopecia interfiere en su vida laboral?<br>0. No, nada. / No, ya no trabajo.<br>1. Sí, un poco.<br>2. Sí, bastante.<br>3. Sí, mucho.
9. ¿Ha dejado de ir a la peluquería o disminuido la frecuencia por la alopecia?<br>0. No.<br>1. Sí, un poco.<br>2. Sí, bastante.<br>3. Sí, mucho.
10. ¿Siente que la gente lo/la mira debido a su alopecia?<br>0. No, nada.<br>1. Sí, un poco.<br>2. Sí, bastante.<br>3. Sí, mucho.
11. ¿Ha abandonado actividades que antes realizaba (ocio, deporte,…) por la alopecia?

0. No, nada.<br>1. Sí, un poco.<br>2. Si, bastante.<br>3. Sí, mucho.
12. ¿Siente que la alopecia interfiere en su vida sexual?<br>0. No, nada.<br>1. Sí, un poco.<br>2. Sí, bastante.<br>3. Sí, mucho.
13. ¿Siente que la alopecia interfiere en su relación de pareja?<br>0. No, nada.<br>1. Sí, un poco.<br>2. Sí, bastante.<br>3. Sí, mucho.
14. ¿Siente que la alopecia interfiere en su relación con sus amistades?<br>0. No, nada.<br>1. Sí, un poco.<br>2. Sí, bastante.<br>3. Sí, mucho.
**Funcional**
15. ¿Pierde tiempo intentando ocultar la alopecia?<br>0. No, nada.<br>1. Sí, un poco.<br>2. Sí, bastante.<br>3. Sí, mucho.
16. ¿Se peina diferente para intentar ocultar la alopecia?<br>0. No, nada.<br>1. Sí, un poco.<br>2. Sí, bastante.<br>3. Sí, mucho.
17. ¿Ha modificado la frecuencia de peinado o lavado de cabello por miedo a incrementar la caída?<br>0. No, nada.<br>1. Sí, un poco.<br>2. Sí, bastante.<br>3. Sí, mucho.
18. ¿Consigue en algún momento olvidar su alopecia?<br>0. Sí, muchas veces.<br>1. Si, bastantes veces.<br>2. Sí, pero pocas veces.<br>3. No, en ninguno.
19. ¿Se pinta las cejas para ocultar el defecto o se las ha tatuado?<br>0. No. / No, no he perdido las cejas.<br>1. Sí, me las pinto ocasionalmente.<br>2. Sí, me las pinto bastantes veces.<br>3. Sí, me las pinto a diario. / Sí, las tengo tatuadas.
20. ¿Utiliza algún tipo de prótesis capilar para disimular su alopecia (peluca, sistema capilar o similar)?<br>0. No, nunca.<br>1. Sí, ocasionalmente.<br>2. Sí, a menudo.<br>3. Sí, siempre.
**Puntuación:** afectación leve (< 21), afectación moderada (21-35), afectación severa (> 35)
